# Monocyte-induced recovery of inflammation-associated hepatocellular dysfunction in a biochip-based human liver model

**DOI:** 10.1038/srep21868

**Published:** 2016-02-23

**Authors:** Marko Gröger, Knut Rennert, Benjamin Giszas, Elisabeth Weiß, Julia Dinger, Harald Funke, Michael Kiehntopf, Frank T. Peters, Amelie Lupp, Michael Bauer, Ralf A. Claus, Otmar Huber, Alexander S. Mosig

**Affiliations:** 1 Institute of Biochemistry II, Jena University Hospital, 07743 Jena, Germany; 2 Department of Anesthesiology and Intensive Care, Jena University Hospital, Jena 07747 Jena, Germany; 3 Institute of Forensic Medicine, Jena University Hospital, 07743 Jena, Germany; 4 Molecular Hemostaseology, Jena University Hospital, Jena, 07747 Jena, Germany; 5 Institute of Clinical Chemistry and Laboratory Diagnostics, Jena University Hospital, 07747 Jena, Germany; 6 Institute of Pharmacology and Toxicology, Jena University Hospital, Jena, Germany; 7 Center for Sepsis Control and Care, Jena University Hospital, Jena, 07747 Jena, Germany

## Abstract

Liver dysfunction is an early event in sepsis-related multi-organ failure. We here report the establishment and characterization of a microfluidically supported *in vitro* organoid model of the human liver sinusoid. The liver organoid is composed of vascular and hepatocyte cell layers integrating non-parenchymal cells closely reflecting tissue architecture and enables physiological cross-communication in a bio-inspired fashion. Inflammation-associated liver dysfunction was mimicked by stimulation with various agonists of toll-like receptors. TLR-stimulation induced the release of pro- and anti-inflammatory cytokines and diminished expression of endothelial VE-cadherin, hepatic MRP-2 transporter and apolipoprotein B (ApoB), resulting in an inflammation-related endothelial barrier disruption and hepatocellular dysfunction in the liver organoid. However, interaction of the liver organoid with human monocytes attenuated inflammation-related cell responses and restored MRP-2 transporter activity, ApoB expression and albumin/urea production. The cellular events observed in the liver organoid closely resembled pathophysiological responses in the well-established sepsis model of peritoneal contamination and infection (PCI) in mice and clinical observations in human sepsis. We therefore conclude that this human liver organoid model is a valuable tool to investigate sepsis-related liver dysfunction and subsequent immune cell-related tissue repair/remodeling processes.

The liver plays a central role in metabolism of carbohydrates, lipids and proteins, in biotransformation and detoxification. In addition, it is a key player in the host response against infections and damage but the multiple impacts of the liver in sepsis just recently started to emerge^1^. Pathogen-associated molecular patterns (PAMPs) inducing systemic inflammatory conditions often result in liver dysfunction as one of the earliest events. They often progress to liver damage and failure and concomitant sepsis-related multi-organ failure (MOF)^2^. Liver injury or damage before onset of sepsis is associated with a higher risk of poor outcome^3^. Yet, the underlying molecular mechanisms are poorly understood. Most studies on inflammation-associated hepatocellular dysfunction were performed in rodent animal models. However, a controversial debate about the value of these models in inflammatory research and transferability of results to human conditions has been raised^4^,^5^. To solve this issue it is necessary to establish model systems that allow corresponding tests under physiological relevant conditions using human cell layers, which clearly mirror the disorganization during the underlying disease in the clinical setting.

The immune response within the liver is mainly borne by nonparenchymal cells (NPCs) accounting for about 40% of total liver cells. Kupffer cells, specialized tissue macrophages, represent 15% of total liver cells and almost 80–90% of all tissue macrophages in the body^6^, underscoring the important role of the liver for detoxification of endotoxin and for immunological homeostasis. In the context of lipopolysaccharide (LPS) stimulation, a recent study suggested that macrophages are key regulators of the inflammatory response in the liver *in vivo*, since upon macrophage-ablation hepatocytes do not longer respond toward a challenge with LPS^7^. Further major cell types populating the liver are stellate cells (6%) and endothelial cells (19%)^8^. Hepatic stellate cells (HSCs) also participate in immune regulation and tissue regeneration^9^. In secreting TNFα and IL-1β they promote an inflammatory microenvironment that aggravates initial damage inflicted by PAMPs and drugs^3^. On the other hand, they also secrete TGF-β and proteins of the extracellular matrix (e.g. collagen I) which leads to resolution of inflammation and tissue repair^10^. The lack of efficient cell depletion strategies for HSCs makes it difficult to assess their respective contribution during inflammation *in vivo*. Similarly, the role of endothelial cells (ECs) comprises not only their barrier function by spatially separating the plasma compartment, but also includes receptor-mediated clearance of endotoxins, bacteria and other compounds, and regulation of inflammation, leukocyte recruitment and host immune responses to pathogens^11^. ECs promote the inflammatory process, by sensing cell debris from damaged hepatocytes via TLR, subsequent secretion of chemokines and expression of cell adhesion molecules that recruit leukocytes to the site of activated and damaged tissue. However, it remains to be elucidated whether they also play a role in recruitment of monocytes that can favor regeneration. Furthermore, the integrity of the liver microvasculature preserving an adequate function of sinusoidal ECs, plays a crucial role in maintaining liver perfusion and liver cell viability^12^.

To investigate human hepatic inflammatory response, primary hepatocyte monolayers are frequently used as they provide the whole range of liver-specific metabolism, including transporter proteins and CYPs relevant in human biotransformation and detoxification. However, primary hepatocytes as well as most of the commonly used hepatocyte cell lines tend to de-differentiate during culture resulting in a loss of metabolic activity and reduced synthesis of typical hepatic products such as urea and albumin^11^. Under physiological conditions, hepatic inflammation and tissue repair in response to microbial infection is orchestrated by the specifically timed and dynamically regulated action of pro- and anti-inflammatory chemo- and cytokines^13^. Such a complex secretion profile can only barely be mimicked by exogenous cytokine treatment. In order to better reflect the *in vivo* situation it is therefore desirable to include an endogenous source of integrative cytokine secretion such as NPCs into the liver model to imitate spatio-temporal events of host response. Several *in vitro* liver models were reported that allow co-culture of hepatocytes with ECs^14^, macrophages^15^ and HSCs^16^.

We recently reported the establishment of a perfused human liver organoid model composed of a vascular and a hepatic cell layer comprising all major cell types of the liver that are assembled in a in tissue structure mimicking the *in vivo* situation^17^. We here adapted the arrangement of the individual organoid cell layers in order to facilitate the enrichment of secreted cytokines, metabolic marker molecules and released intracellular enzymes in response to TLR-mediated inflammation. We observed a striking resemblance to the sepsis-related liver dysfunction responses in the well-established *in vivo* mouse model of peritoneal contamination and infection (PCI) as well as clinical observations of human sepsis^2^. To assemble the liver organoid we used the recently described microfluidically supported “MOTiF” (multi organ tissue flow) biochips^18^ enabling adhesion and transmigration of monocytes under physiological flow conditions to study the impact of invading immune cells on the functional properties of the liver organoid under inflammatory conditions. Strikingly, we observed a restoration of inflammation-induced hepatocellular dysfunction upon adhesion of monocytes to the vascular layer and their invasion into inflamed hepatic tissue. Thus, this model not only mimics inflammation-related processes of liver dysfunction on the cellular level, but also allows analysis of monocyte-mediated processes of tissue repair and immunotolerance known to be important in the course of human sepsis^19^.

## Results

### Design of the biochip-based sinusoidal liver model

The structure of the artificial liver model was inspired by the structure of the human liver sinusoid. A suspended porous membrane within the MOTiF biochips served as a substrate for human umbilical vein endothelial cells (HUVEC) that are co-cultured with primary macrophages mimicking the immune-modulatory function of Kupffer cells. The EC/macrophage layer mimics aspects of the vascular component of the human liver sinusoid in shielding hepatocytes from instant contact to monocytes perfused via the upper channel system thereby imitating the luminal side of the sinusoid. This vascular layer is separated by a medium-filled space of the size of 400 μm from the hepatic cell layer cultured on the sealing foil at the bottom of the biochip. The hepatic layer comprises human HepaRG hepatocytes that differentiate into cells with a hepatocyte phenotype and cells exhibiting a biliary epithelial cell phenotype which self-organize during culture and form hepatocyte layers with functional bile canaliculi^20^. This design allows to also address the important role of the biliary epithelium within the liver. Furthermore, HepaRG cells have been shown to be suitable for studies of hepatic inflammatory response^21^,^22^.

HepaRG cells were co-cultured with HSCs that *in vivo* mostly reside in the space of Dissé with contacts to both endothelial cells and hepatocytes^23^. To keep the endothelial layer intact LX-2 stellate cells were co-cultured in the hepatic layer. This hepatic layer separated from the vascular layer by superposed medium and the membrane allows cross-communication of both layers and cell migration through the vascular to the hepatic tissue layer through the pores of the membrane (*SI Appendix*, Fig. S1A,B).

### NPCs stabilize HepaRG differentiation in the absence of dimethyl-sulfoxide

HepaRG cells were used to establish the liver organoid as this cell line in contrast to other hepatic cells lines, i.e. HepG2 or Hep2/C3A, remains functionally stable during prolonged culture and self-organizes functional bile canaliculi-like structures^24^. Moreover, HepaRG cells respond to exogenous inflammatory cytokine treatment^25^. Maintenance of differentiated HepaRG cells depends on the presence of 2% dimethyl-sulfoxide (DMSO) and is a prerequisite for the expression of cytochrome P450 enzymes (CYP proteins), which is diminished in its absence (*SI Appendix*, Fig. S1C). However, during prolonged cell culture DMSO is cytotoxic to HUVEC cells forming the vascular layer. They dramatically lose expression of endothelial von Willebrand factor and PECAM-1, proteins that are involved in regulation of coagulation, immune cell trafficking and maintenance of EC junctional integrity^26^,^27^. Co-culture of NPCs has been shown to stabilize hepatocyte differentiation^28^. Indeed, when differentiated HepaRG cells were co-cultured with LX-2 stellate cells, HUVEC and macrophages prevented HepaRG dedifferentiation resulting in stabilization of CYP3A4 expression for at least four days of co-culture even in the absence of DMSO (*SI Appendix*, Fig. S1C). From these observations we concluded that co-culture of NPCs stabilizes the differentiated hepatocyte phenotype in the liver organoid.

### TLR agonist-specific cytokine secretion profiles

**Figure 1:**
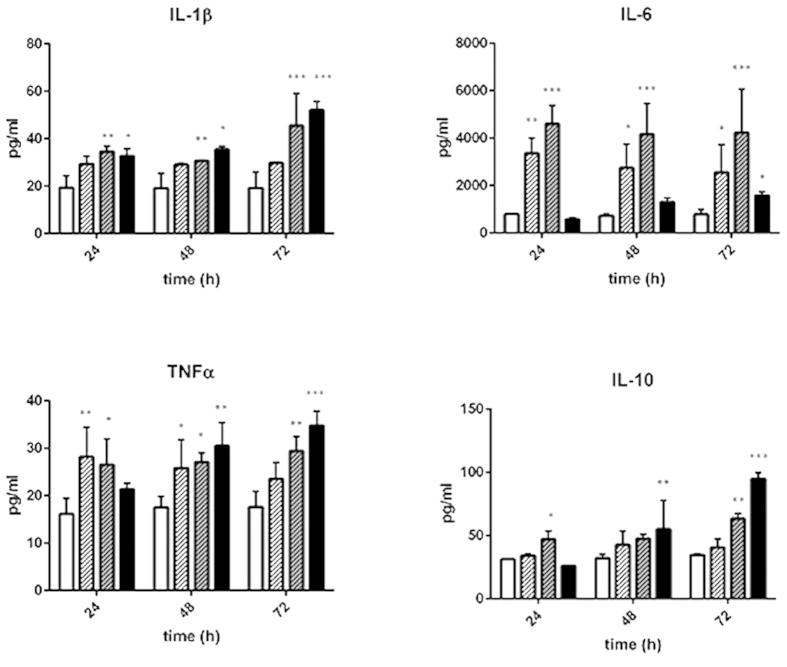
Cytokine profiling of liver organoids stimulated with TLR agonists. Cytokine release of untreated liver organoids (open bars) was compared to liver organoids treated for 24 h, 48 h or 72 h with Pam3CSK4 (open, shaded bars), LPS (grey, shaded bars) or ODN2006 (black bars). Cell culture medium was exchanged every 24 h. Statistical significance was calculated compared to untreated control after 24 h of culture using two-way ANOVA with Dunnett’s multiple comparisons test (*p < 0.05, **p < 0.01, ***p < 0.001). Results of five independent experiments are shown.

In a first set of experiments, the inflammatory response of the liver organoid was tested in response to treatment with Pam3CSK4 (TLR-1/2 agonist), LPS (TLR-4 agonist) and ODN2006 (TLR-9 agonist) for up to 72 h. A TLR agonist-specific and time-dependent cytokine secretion profile of the pro-inflammatory cytokines IL-1β, IL-6 and TNFα and the anti-inflammatory cytokine IL-10 was observed. The release of pro-inflammatory cytokines occurred as an acute and sustained pro-inflammatory response for up to three days of stimulation. The release of anti-inflammatory IL-10 was delayed and only induced by LPS and ODN2006 treatment, reaching a maximum after 72 h of stimulation (Fig. 1)

### Hepatic enzyme expression is modulated by TLR stimulation

**Figure 2:**
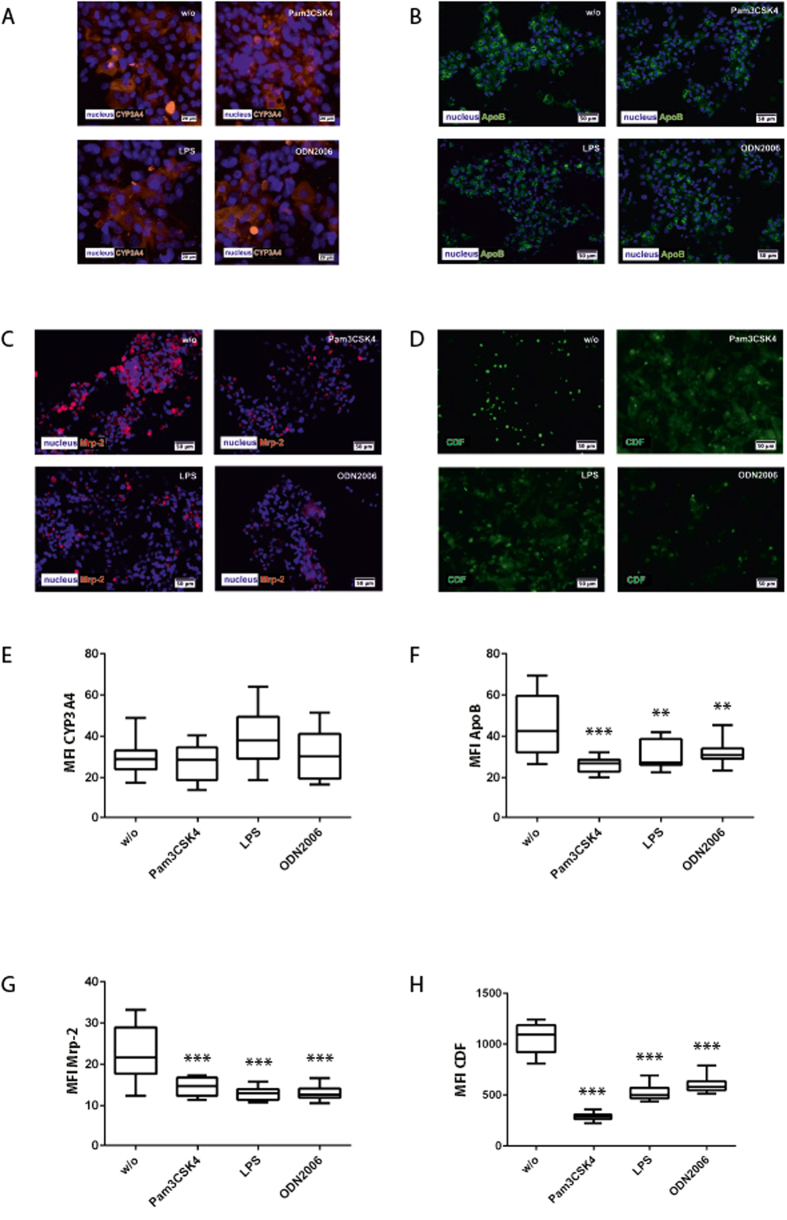
Hepatocyte protein expression and CDF secretion in the hepatic layer. Liver organoids were treated with Pam3CSK4, LPS or ODN2006 for 72 h and compared with untreated control (w/o). (**A**–**D**) Immunofluorescence staining of (**A**) CYP3A4 (orange) (**B**) ApoB (green) and (**C**) MRP-2 (magenta), (**D**) CDF secretion (green) into bile canaliculi after 24 h of stimulation with TLR agonists. (**E–H**) Computational analyses of fluorescence signal intensities using random field analyses of at least 20 regions of interest (ROI) per tested condition (labeled as mean immunofluorescence intensity (MFI) of specific staining against the respective protein) of (**E**) CYP3A4, (**F**) ApoB, (**G**) Mrp-2, (**H**) CDF. Nuclei are stained with DAPI (blue). Statistical significance was calculated compared to untreated control using one-way ANOVA with Dunnett’s multiple comparisons test (*p < 0.05, **p < 0.01, ***p < 0.001). Results of three independent experiments are shown.

CYP3A4 as one of the major cytochrome P450 enzymes is responsible for the metabolism of >50% of all prescribed drugs^29^. In accordance with previous reports^21^,^30^ we found a down-regulation of CYP3A4 in response to LPS stimulation in mono cell cultures of HepaRG cells. A significant reduction of CYP3A4 expression in response to LPS was also observed in co-cultures of HepaRG with stellate cells (*SI Appendix*, Fig. S2A). Interestingly, at the level of a completely assembled liver organoid including the endothelial layer and macrophages the expression of CYP3A4 was not affected by any of the TLR agonists (Fig. 2A,E). To correlate expression and drug-metabolizing activity we used midazolam as CYP3A4 model substrate and measured the formation rate of its metabolite 1-OH-midazolam over 6 h in HepaRG mono-cell cultures and in liver organoids. Although LPS treatment diminished formation rate of 1-hydroxymidazolam (1-OH-midazolam) in HepaRG mono-cell cultures we observed no significant decline in response to LPS stimulation in the liver organoid (*SI Appendix*, Fig. S2B). These data suggested that ECs and macrophages of the vascular layer contribute to a reduced susceptibility of the hepatocyte response to LPS in respect to CYP3A4 activity.

We further asked whether the liver organoid reflects the inflammation-related lipoprotein dysregulation at the level of apoB expression. In agreement with *in vivo* studies^31^, we observed a significantly reduced expression of apoB in the liver organoids after treatment with agonists for TLR1/2, TLR4 and TLR9 (Fig. 2B,F).

Moreover, sepsis-related liver dysfunction is associated with cholestasis^32^. Expression and activity of MRP-2, a major transporter protein involved in bile acid secretion, was used as a read-out. Release of the fluorescent dye 5 (and 6)-carboxy-2′,7′-dichlorofluorescein (CDF) into bile canaliculi was measured as surrogate for MRP-2 efficiency^33^. After 72 h, expression levels of MRP-2 remarkably declined in liver organoids treated with Pam3CSK4, LPS or ODN2006 compared to untreated control (Fig. 2C,G). Moreover, upon TLR stimulation accumulation of CDF in the bile canaliculi was substantially reduced and CDF was trapped within the cytoplasm of the hepatocytes (Fig. 2D,H).

### Inflammatory response of the vascular layer

**Figure 3:**
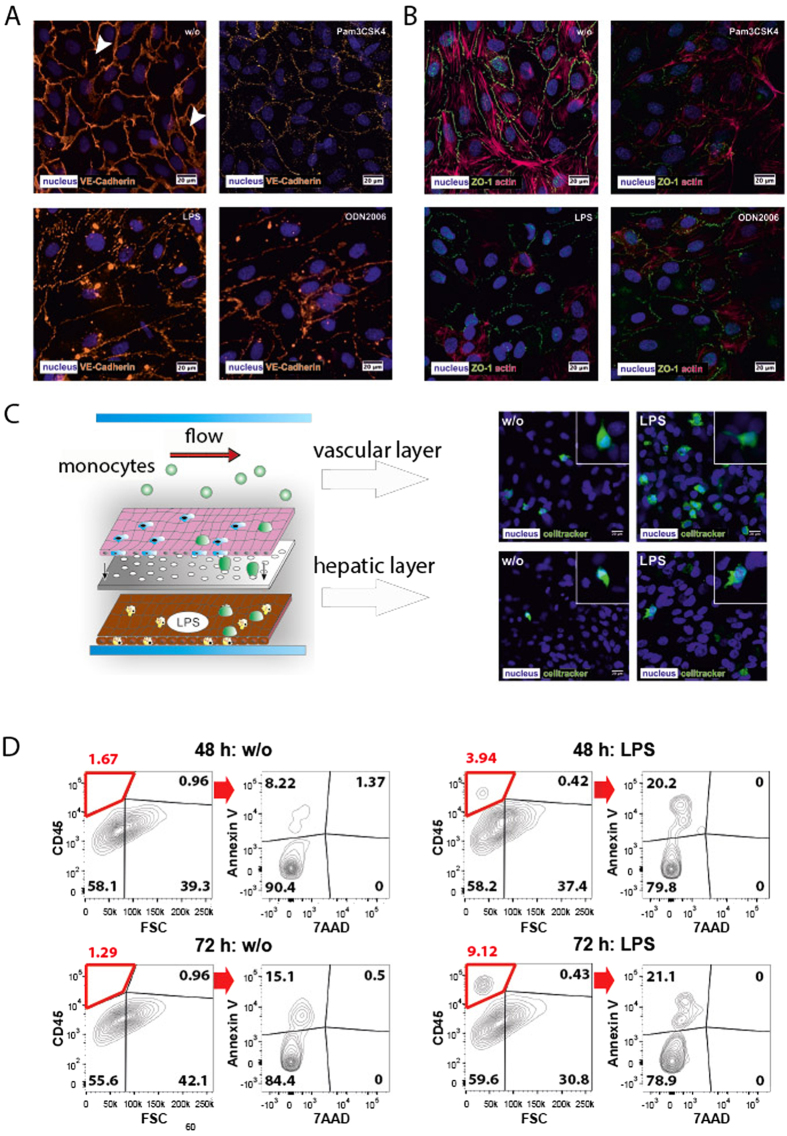
Modulation of endothelial integrity and monocyte adhesion/migration under inflammatory conditions triggered by Pam3CSK4, LPS or ODN2006. (**A**) Expression of VE-cadherin (orange). White arrows indicate gaps in the vascular layer. (**B**) Expression of ZO-1 (green) and actin (red). (**C,D**) Flow-based adhesion and migration assay of primary monocytes stained with Celltracker^®^ Green at the vascular and hepatic layer of untreated liver organoids (w/o) or liver organoids pre-stimulated with LPS. Nuclei are stained with DAPI (blue). (**D**) Analysis of primary monocytes transmigrated for 48 h and 72 h into the hepatic layer in absence (w/o) or presence of LPS. Monocytes within the hepatic chamber were stained and gated based on CD45 expression and analyzed for apoptosis induction (Annexin V staining) and cell death (7AAD). (**A–D**) Results of a representative experiment out of three independent experiments are shown.

During sepsis a dysregulation of the endothelial barrier is a major pathogenic mechanism promoting septic MOF^34^. Therefore, we next studied the response of ECs and embedded macrophages to TLR agonists in the liver organoid. Both cell types have been shown to bind LPS and mediate endotoxin clearance in the liver^35^. Expression and localization of the junctional proteins VE-cadherin and the zonula occludens-1 (ZO-1) protein in the vascular layer were analyzed in response to inflammation. VE-cadherin is known to be essential for endothelial integrity and the maintenance of endothelial cell-cell contacts^36^. ZO-1 connects integral tight junction proteins to the actin cytoskeleton, and was reported to be involved in the disarrangement of the actin cytoskeleton leading to increased endothelial permeability in response to IL-6^37^. All TLR agonists tested induced a sustainable reduction of VE-cadherin and ZO-1 expression within three days of stimulation (Fig. 3A,B). Concomitantly, actin cytoskeleton fibers within ECs were disassembled in response to TLR stimulation (Fig. 3A,B).

Endothelial injury is associated with the recruitment of circulating immune cells such as monocytes to the endothelial lining, where they are assumed to contribute to local inflammatory hotspots and thereby enhance endothelial leakage. Since in initial experiments LPS provoked a robust and prolonged pro-inflammatory cytokine release among tested TLR agonists, we focused our studies on monocyte adhesion and transmigration into the liver organoid in response to LPS treatment. To assess specific cell recruitment and diapedesis at the vascular layer under flow conditions, liver organoids pre-cultured and stimulated under static conditions were subsequently perfused with Celltracker^®^ Green stained monocytes for 60 min under dynamic flow conditions with a shear stress rate of 3 dyn/cm^2^. Primary monocytes as well as THP-1 monocytes were efficiently recruited to the vascular layer upon LPS stimulation, formed filopodia and efficient diapedesis through the vascular layer and migration into the hepatic compartment of the liver organoid was observed (Fig. 3C,D; *SI Appendix*, Fig. S3A). Viability of transmigrated primary monocytes of more than 75% was confirmed by flow cytometry (Fig. 3D). These findings prove that the majority of invading monocytes in the liver model were viable and functionally active in respect to adhesion and endothelial transmigration.

Monocyte adhesion/migration enhances the cytokine release, which activates endothelial cells and thereby induces the expression of ICAM-1 and VCAM-1 and release of sCAMs^38^. Immune cell adhesion and subsequent migration rely on the presence of endothelial cell adhesion molecules (CAMs) whereas release of soluble CAM fragments was shown to correlate with severity of sepsis^39^. An acute increase of endothelial ICAM-1 and VCAM-1 expression in the vascular layer of the liver organoid was observed within 24 h of LPS stimulation. Although ICAM-1 levels went down within the next 48 h, expression was still considerably higher than in unstimulated cells. By contrast VCAM-1 expression returned to basal levels. Interestingly, in the presence of perfused and adhesive monocytes, ICAM-1 expression on EC surfaces was significantly lower under inflammatory conditions triggered by LPS after 72 h compared to LPS-treated organoids without monocyte perfusion. VCAM-1 levels in monocyte-perfused organoids were slightly lower compared to monocyte-free conditions (*SI Appendix*, Fig. S3A).

**Figure 4:**
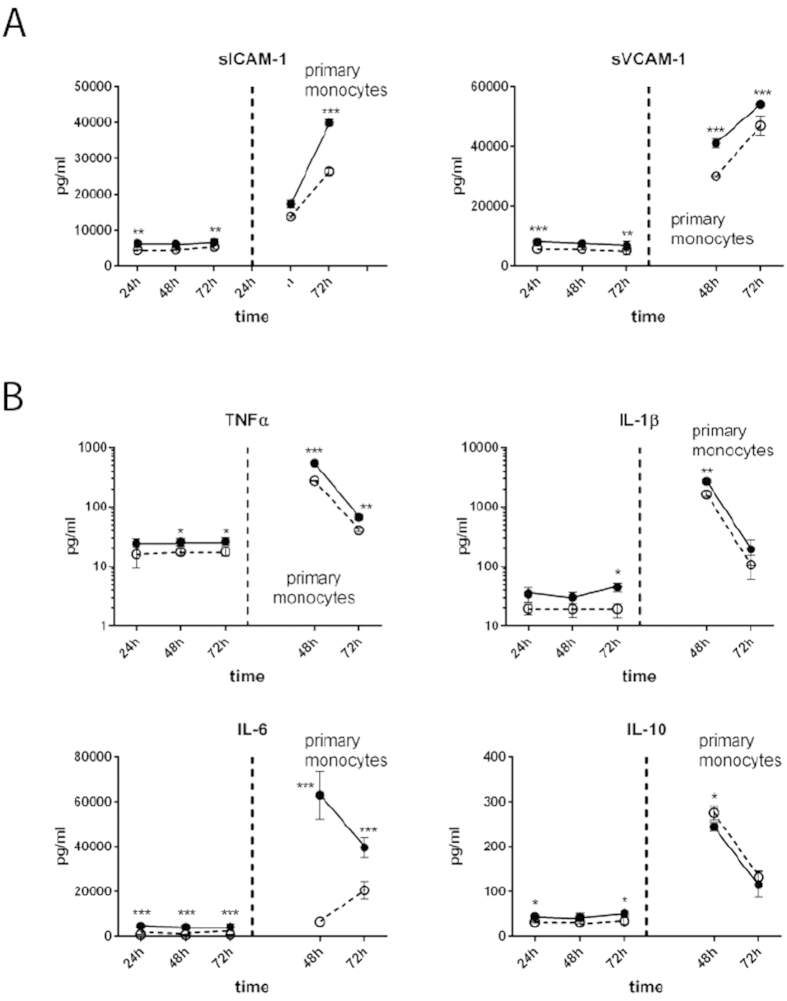
Impact of perfusion with primary monocytes. (**A**) Release of sICAM and sVCAM in response to primary monocyte perfusion and adhesion. (**B**) Secretion of IL-1β, IL-6, TNFα and IL-10 in response to primary monocyte perfusion and adhesion. Liver organoids were untreated (dashed line) or stimulated with LPS (solid line), without monocyte perfusion (left from vertical dashed line) or with monocyte perfusion (right from vertical dashed line). (**A**,**B**) Statistical significance was calculated between untreated and LPS-treated liver organoids of identical time points and perfusion conditions (*p < 0.05, **p < 0.01, ***p < 0.001) using student’s t-test. Results of six independent experiments are shown.

LPS-treatment induced a prompt release of sICAM-1 and sVCAM-1 from the cell surface within 24 h. Within the next 48 h sICAM-1 levels only slightly increased whereas sVCAM-1 levels decreased. In the presence of monocytes sCAM levels decreased (Fig. 4A). As expected, we further found increased IL-1β, IL-6 and TNFα levels after primary monocyte adhesion in the presence of LPS compared to untreated control. Cytokine levels then declined during subsequent 48 h, but remained significantly higher in LPS-treated liver organoids (Fig. 4B). Also anti-inflammatory IL-10 was regulated in a similar manner in primary monocytes.

In liver organoids perfused with THP-1 monocytes we found a significantly reduced release of cytokine upon LPS challenge and TNFα release was not increased in presence of THP-1 monocytes. In addition, a transient increase of anti-inflammatory IL-10 secretion was detectable in untreated liver organoids and to lower extend in LPS-treated organoids in the presence of THP-1 monocytes (*SI Appendix*, Fig. S4B). In contrast to primary monocytes the release of sICAM and sVCAM was not significantly increased by LPS in the presence of THP-1 monocytes (*SI Appendix*, Fig. S3C).

### Invading monocytes modulate endothelial and hepatic protein expression and function in the liver organoid

**Figure 5:**
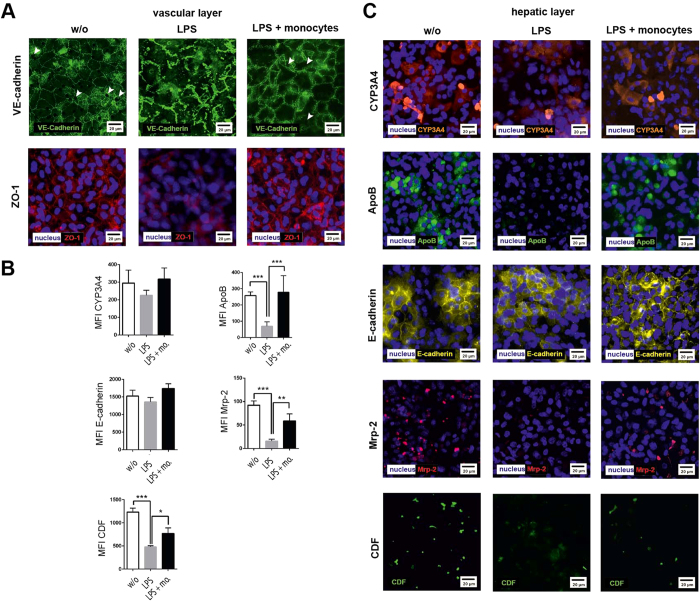
Immunofluorescence staining of endothelial and hepatocyte proteins without stimulation (w/o) and in presence of 100 ng/ml LPS (LPS) or 100 ng LPS and primary monocytes (LPS + monocytes (mo.)). (**A**) Expression of VE-cadherin and ZO-1 at the vascular layer. White arrows indicate gaps in the vascular layer. Representative results of three independent experiments are shown. (**B**) Computational analyses of fluorescence intensities of at least 20 ROI per condition (labeled as mean immunofluorescence intensity (MFI)) of the respective protein using random field analysis in the hepatic layer. Statistical significance was calculated between indicated conditions using student’s t-test (*p < 0.05, **p < 0.01, ***p < 0.001). Immunostaining for (**C**) CYP3A4 (red), ApoB (green), E-cadherin (yellow), MRP-2 (red), and detection of CDF secretion (green). Nuclei are stained with DAPI (blue). Representative results of three independent experiments are shown.

In LPS stimulation experiments without monocyte perfusion, localization of VE-cadherin and ZO-1 appeared fuzzy and discontinuously aligned along cell-cell contacts. In contrast, only a minor loss of VE-cadherin expression accompanied with an enhanced cytosolic VE-cadherin signal was found within the vascular layer in the presence of adhering/invading primary monocytes in response to 72 h LPS treatment (Fig. 5A).

To characterize the impact of invading monocytes on the hepatic cells, we also measured the expression of CYP3A4, ApoB, MRP-2 and E-cadherin within the hepatic layer. In experiments without monocyte perfusion we observed that LPS treatment did not affect CYP3A4 or E-cadherin expression but induced a decrease of ApoB and MRP-2 expression accompanied by a reduced MRP-2 activity in bile canaliculi under inflammatory conditions (Fig. 5B). Importantly, in the presence of invading monocytes we observed a stabilization of ApoB and MRP-2 expression associated with the secretion of CDF. Although the expression level of these proteins as well as the CDF secretion rate were lower compared to naive organoids, invading monocytes clearly prevented hepatocellular dysfunction observed under inflammatory conditions in absence of migrating monocytes. Similar observations were made upon THP-1 invasion in presence of LPS (*SI Appendix*, Fig. S5A,B).

**Figure 6:**
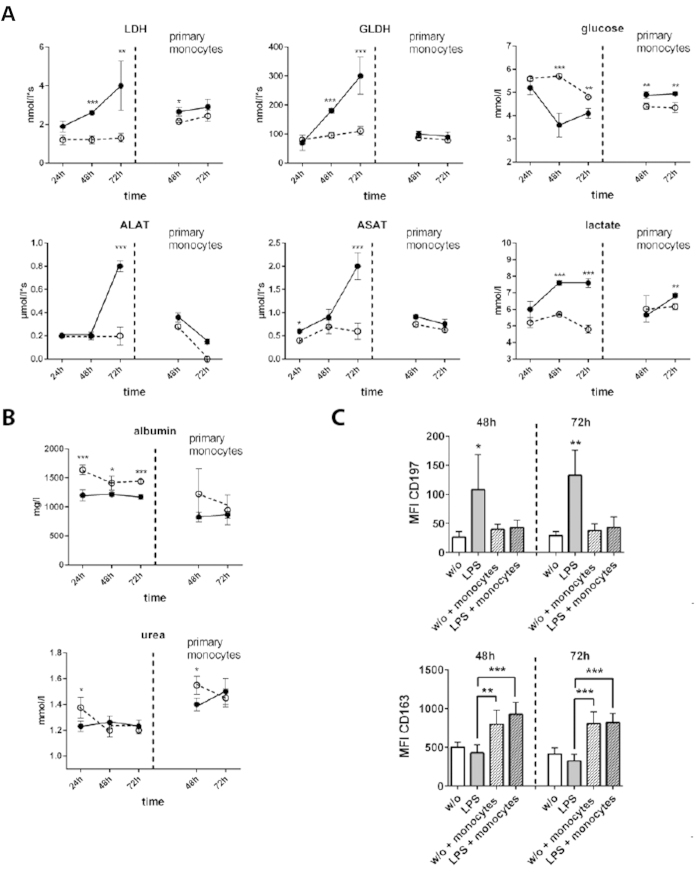
Release of intracellular enzymes and metabolic activity of the liver organoid. Liver organoids were untreated (dashed line) or stimulated with LPS (solid line), without monocyte perfusion (left from vertical dashed line) or with primary monocyte perfusion (primary monocytes, right from vertical dashed line). (**A**) Release of lactate-dehydrogenase (LDH), glutamate-dehydrogenase (GLDH), aspartate-transaminase (ASAT) and alanine-transaminase (ALAT). Changes in glucose, lactate consumption. (**B**) Synthesis of albumin and urea. Statistical significance was calculated between untreated and LPS-treated samples at similar time points and perfusion conditions (*p < 0.05, **p < 0.01, ***p < 0.001) using student’s t-test. (**C**) Computational analyses of fluorescence intensity of 20 ROI per condition (labeled as mean immunofluorescence intensity (MFI)) of CD197 or CD163 using random field analyses of macrophages in the vascular layer. Liver organoids were cultured for 48 h or 72 h in absence (w/o) or presence of LPS (LPS). Where indicated liver organoids were perfused with monocytes (+monocytes) 24 h after culture and then sub-cultured for indicated times. Significance of CD197 was calculated for condition “LPS treatment without monocytes perfusion” compared to the remaining conditions. For CD163 significance was calculated between indicated conditions. For statistical analysis of CD196 and CD163 expression one-way ANOVA with Bonferroni multiple testing correction (*p < 0.05, **p < 0.01, ***p < 0.001) was used. (**A**–**C**) Data of three independent experiments are shown.

Next, the impact of monocyte invasion into the liver organoid on viability and metabolic activity of hepatic cells was tested. In this context, the release of intracellular enzymes as reporters of cell-damage in response to LPS-induced inflammation was measured. In the absence of monocytes, a continuously increasing release of the intracellular enzyme lactate-dehydrogenase (LDH) under inflammatory conditions was observed, which was not detectable under LPS-free conditions. Most interestingly, adhesion and invasion of primary monocytes as well as THP-1 monocytes resulted in a rapid decline of LDH-release down to levels observed in the untreated control. Similar results were obtained for the release of the hepatocyte-specific intracellular enzymes glutamate-dehydrogenase (GLDH), aspartate-transaminase (ASAT) and alanine-transaminase (ALAT) with increasing release of GLDH, ASAT and ALAT in response to LPS-triggered inflammation. However, in the presence of transmigrated primary as well as THP-1 monocytes, accumulation of GLDH, ASAT and ALAT in the culture medium of the hepatocyte layer was prevented (Fig. 6A, *SI Appendix*, Fig. S6A).

The metabolic activity of the liver organoid was analyzed at the level of cellular glucose consumption and lactate formation. Liver organoids stressed by inflammation exhibited an increased turnover/consumption of glucose compared to non-stimulated liver organoids. Interestingly, in the presence of transmigrated primary monocytes, inflammatory conditions apparently influenced glucose turnover rates to a lower extent. Concomitant with increased glucose consumption, LPS treatment induced an increase in lactate formation. This effect was diminished in the presence of primary monocytes (Fig. 6B), but not observed for THP-1 monocytes (*SI Appendix*, Fig. 6B).

In addition, we studied albumin and urea synthesis under inflammatory conditions in the liver organoid. Under unstimulated conditions, liver organoids stably synthesized albumin and urea. However, inflammation induced by LPS resulted in a drop in albumin secretion. This effect was not observed for urea production, which appeared to be unaffected by LPS treatment. In presence of primary monocytes, the impact of LPS treatment was abolished 48 h after monocyte invasion and urea synthesis rate even increased (Fig. 6B). In the presence of transmigrated THP-1 monocytes, a significant decline of albumin and urea formation was detected within the first 24 h. However, 48 h after monocyte perfusion, albumin and urea production returned back to levels typically seen at untreated conditions even in the presence of LPS (*SI Appendix*, Fig. 6B).

**Figure 7:**
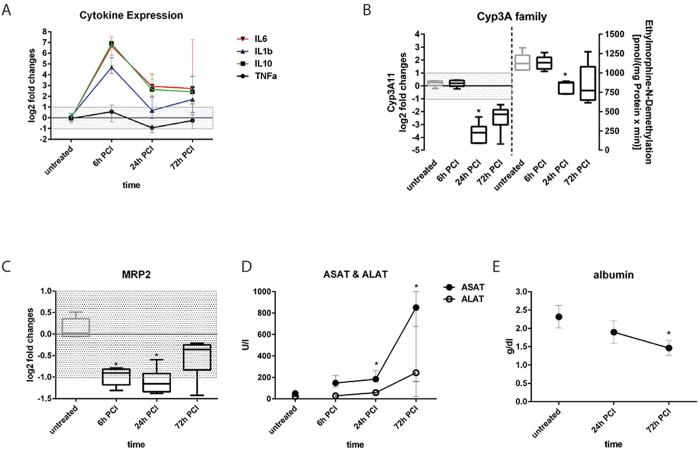
Release of cytokines and intracellular enzymes, and regulation of CYP3A and MRP2 in the PCI model. Messenger RNA expression of cytokines (**A**), CYP3A11 (**B**) and MRP-2 (**C**). Enzyme activity of CYP3A family members using the model reaction ethylmorphine-N-demethylation (**D**,**E**) Serum concentrations of ASAT, ALAT and albumin. Statistically significance was calculated using one-way ANOVA with Dunn’s test for multiple testing correction (^#^p < 0.05). (**A**–**E**) Five animals per time point were analyzed.

To investigate the impact of LPS and migrating monocytes on the activation of macrophages residing in the vascular layer we studied their polarization stage. Resident macrophages were identified by staining the macrophage marker protein CD68^40^ and were analyzed for the expression of M1 polarization marker protein (CCR7) and M2 polarization marker protein CD163 (hemoglobin-haptoglobin complex receptor)^41^. We observed an LPS-induced up-regulation of CD197 48 h and 72 h after LPS stimulation, which was prevented in the presence of migrating monocytes. In contrast expression of CD163 was not influenced by LPS but up-regulated in presence of monocytes (Fig. 6C and *SI Appendix*, Fig. 7.). Our data thus indicate a shift from M1 to M2 polarization of macrophages in the presence of LPS upon monocyte migration.

### Comparison with the *in vivo* situation in a peritoneal contamination and infection mouse model

Next, we wanted to address whether effects in the liver organoid are similarly detectable in the *in vivo* situation. Murine peritoneal contamination and infection (PCI) is a well-established *in vivo* model for studies on systemic inflammation, sepsis-related liver dysfunction and its long-term sequelae with respect to tissue repair^42^,^43^,^44^,^45^. Similar to findings in the liver organoid, cytokine levels rapidly increased in the mouse model within 6 h after induction of systemic inflammation by PCI, however, dropped down to low levels until 72 h at the mRNA level (Fig. 7A). In agreement with other reports we observed a down-regulation of the mRNA levels of CYP3A11, which encodes the human analog of CYP3A4, in response to LPS. In a time series for up to 72 h a maximal repression of the mRNA expression was found 24 h after PCI. Although, gene expression of CYP3A11 appeared to be reduced on the mRNA level as shown by qRT-PCR, a reduction of enzyme activity in response to PCI was only detectable 24 h after PCI (Fig. 7B). In line with our findings in the liver organoid also MRP-2 mRNA expression in the PCI mouse model was down-regulated for up to 24 h after PCI (Fig. 7C). In addition, serum levels of ASAT and ALAT were significantly increased upon PCI induction. Maximal release of ASAT/ALAT was observed three days after PCI, indicating most acute inflammation-related liver damage at this time point (Fig. 7D). Furthermore, albumin production in the PCI mouse model was significantly decreased with a maximal depression of albumin synthesis three days post PCI procedure (Fig. 7E).

## Discussion

Using different protocols to stimulate inflammation we have demonstrated the suitability of our biochip supported liver organoid model to simulate sepsis-related liver dysfunction *in vitro*. Most notably, our studies demonstrated that co-culture of the different cell types of the liver is essential to obtain responses reflecting the *in vivo* situation. In detail, we observed that depending on the TLR agonist used for stimulation a specific and time-dependent profile of pro- and anti-inflammatory cytokines was released. This observation underlines that a complete inflammatory response within the liver not only relies on certain isolated cytokines often used as exogenous stimuli especially in cell culture approaches. In particular, it depends on a concerted action of various pro- and anti-inflammatory activities in response to the PAMP stimulus. The inclusion of NPCs and their intrinsic immune-modulatory capacity into the biochip seems to better reflect inflammatory conditions occurring in human liver.

Under inflammatory conditions we did not observe any reduction of CYP3A4 expression in the liver organoid nor reduced formation of 1-OH-midazolam in response to LPS stimulation. In the PCI mouse model expression and activity of CYP3A11 (the homolog of human CYP3A4) were initially reduced and subsequently increased over time in surviving mice. On the other hand, a down-regulation of CYP3A4 protein expression in primary human hepatocyte culture after stimulation with LPS or pro-inflammatory cytokines (5 ng/ml IL-1 or 10 ng/ml TNFα) was reported^46^. The differences to our observations can be explained by the fact, that in our study a significantly lower dose of LPS was used (100 ng/ml vs. 10 μg/ml LPS). Furthermore, a significantly different amount of secreted cytokines was measured in the liver organoid even in the presence of invading monocytes (IL-1β: 516 ± 16 pg/ml and TNFα: 29 ± 3 pg/ml) compared to more than 9-fold and 340-fold higher concentrations of IL1β or TNFα used in the study of Aitken *et al.*^46^. Thus endogenous cytokine release by the organoid model could avoid stimulation with overdosed cytokine amounts potentially inducing non-physiological responses.

Moreover, the response of the liver organoid to TLR agonists resembles many metabolic and structural changes reported for septic liver dysfunction and damage including down-regulation of ApoB, MRP-2, albumin and increased release of LDH, GLDH, ALAT and ASAT. It has been shown that serum ApoB levels are significantly reduced in patients suffering from a systemic inflammatory response syndrome with associated MOF^31^. Furthermore, it was reported that non-survivors of severe sepsis exhibit diminished serum LDL levels compared to surviving patients^47^. Also MRP-2 expression and function was shown to be restricted during sepsis. In the course of sepsis, MRP-2 is irregularly expressed and disrupted at sides of hepatocellular bile secretion in rats, resulting in a disturbed bile acid transport and secretion^2^. In addition, our experiments suggest that endothelial cells actively participate in the regulation of liver organoid inflammation. Up-regulation and shedding of ICAM-1 and VCAM-1 support observations that enrichment of sCAM fragments in the serum positively correlates with the severity of sepsis and other systemic inflammatory diseases^39^,^48^.

It has been shown that NPCs are the major source of inflammatory cytokines such as IL-6 driving acute phase protein production in hepatocytes^49^. HSCs express TLR4 and TLR9, and in addition, they can secrete potent chemoattractants for macrophages (Mcp-1, RANTES) and neutrophils (Cxcl1/Gro1)^50^. In the liver organoid primary as well as THP-1 monocytes were recruited to the activated vascular layer in response to LPS treatment. Monocyte invasion into the liver organoid triggered by PAMP-induced inflammation resulted on one hand in an increased release of pro-inflammatory cytokines such as IL-1β and IL-6, but on the other side appeared to contribute to a dampening of the inflammation-induced hepatocellular dysfunction. However, variations in the monocyte-mediated modulation of liver organoid function under inflammatory conditions were observed when primary and THP-1 monocytes are compared. It is known that THP-1 monocytes secrete substantially lower amounts of TNFα, IL-6 and IL-10 upon LPS stimulation^51^ compared to primary monocytes, which was also detectable in our LPS-stimulated and monocyte-perfused liver organoids. Thus, this different behavior likely explains the observed variations of endothelial ICAM-1 expression and sICAM-1 release upon LPS treatment as these cytokines are critical regulators of ICAM-1 expression and shedding^52^,^53^. However, we already observed an increased cytokine release in the presence of primary monocytes that was associated with an augmented sICAM/sVCAM shedding in the absence of LPS. This might be a reflection of a higher stimulatory sensitivity of primary monocytes compared to THP-1 cells when they are perfused in the vascular compartment of the liver organoid.

Although expression of ApoB and MRP-2 as well as efficiency of MRP-2-mediated bile acid secretion remained decreased during time of observation, the LPS-mediated decrease in albumin or urea production as well as a nearly complete abolishment of hepatic enzyme release into the cell culture medium upon monocyte invasion was observed. This fact indicates that the presence of monocytes within inflamed liver tissue may contribute to increased survival of the tissue layers of the liver organoid.

Macrophages have also been shown to mediate tissue regeneration in response to drug-induced liver damage depending on their differentiation stage^54^,^55^. M1 or classically activated macrophages possess pro-inflammatory properties and are triggered by bacterial molecular components such as LPS. M2 or alternatively activated macrophages, are generated in response to cytokines such as IL-10 and have been shown to mediate tissue regeneration^56^. The polarization of tissue macrophages is thereby not a terminal state but a transient polarization status within a continuum of different polarization stages with sometimes overlaying macrophage polarization phenotypes. Based on evaluation of the expression pattern of the M1 and M2 polarization markers CD197 and CD163, respectively, we observed a shift from LPS-induced M1- to M2-polarization upon monocyte invasion. The up-regulated expression of the M1 marker protein CD197 in macrophages of the vascular layer in the liver organoids is likely to be induced by LPS and was described in several studies^41^. Upon monocyte perfusion we found a significant increase of IL-10 release, a cytokine known to facilitate M2-polarization^57^. We speculate that the significant increase of IL-10 secretion contributes to the observed polarization shift upon primary monocyte invasion. This hypothesis is supported by the observation that up-regulation of the M2-polarization marker CD163 correlates with the release of IL-10 and primary monocyte migration but not with LPS treatment. In addition to increased IL-10 release monocyte migration further increases the phagocytic fraction in the liver organoid. This likely facilitates clearance of soluble LPS by monocytes infiltrating the organoid, which facilitates removal of the primary M1-polarization trigger. In combination these processes can contribute to the observed macrophage polarization shift associated with the restoration of organoid function. In addition to macrophage polarization, a immunosuppressive state of monocytes due to cellular re-programming associated with tissue re-modeling and wound healing was described in sepsis patients^58^,^59^. Shalova *et al.* recently showed that monocytes of patients suffering from sepsis displayed a pro-inflammatory gene expression profile that was associated with features of endotoxine tolerance when treated *ex-vivo* with LPS^59^. Still, phagocytosis and anti-microbial activity as well as tissue re-modeling and repair functions were found to be increased in blood monocytes of sepsis patients, indicating that monocyte function is re-configured under these conditions^59^. Similar effects were also observed in our liver organoid model, where after an initial acute pro-inflammatory phase further release of pro-inflammatory cytokines was subsequently suppressed. This effect was not observed in absence of invading monocytes where cytokine release was stable up to 72 h of LPS stimulation. From our data, we speculate that a homeostatic situation is established in the liver organoid within 48 h of co-culture with transmigrated monocytes facilitating on the one hand inflammation, and on the other hand mediating endotoxin clearance, immune tolerance as well as enhanced metabolism and viability of hepatocytes.

Recently, Esch *et al.* reported the establishment of a three-dimensional model of the human liver, consisting of defined ratios of hepatocytes and NPCs (fibroblasts, stellate cells, and Kupffer cells)^60^. In this model, stable secretion of albumin and urea, and responsiveness to LPS was shown as detected by secretion of pro-inflammatory IL-8. However, this model did not include an endothelial counterpart. Furthermore, Prodanov *et al.* published a model consisting of collagen-gel-embedded hepatocyte/stellate cell co-cultures which are separated by a porous membrane from a cell layer of endothelial like HUVEC/A549 somatic cell hybrids co-cultured with U937 cells^61^. In a cursory test for its inflammatory response, a stable expression of CYP3A4 was observed in primary hepatocytes co-cultured with non-parenchymal cells similar to our model. Though, to the best of our knowledge, we herein describe for the first time a microfluidically assisted human liver sinusoid model specifically designed to mimic responses to liver inflammation that not only exhibits typical signs of pathophysiological liver dysfunction, but also reflects monocyte-mediated tissue repair and reduction of inflammation-triggered cell death.

Taken together, our biochip-based liver organoid exhibits highly similar responses to inflammatory stimulation as compared to those in mice undergoing sepsis. In absence of invading monocytes we observed an acute response ultimately resulting in hepatocellular dysfunction and cell death. However, circulating monocytes recruited to the vascular layer in response to inflammation that subsequently migrated into the stimulated organoid seem to act as modulators mediating different early and late inflammatory responses. Monocytes abrogated inflammation-related cell death and induced recovery of central metabolic functions in liver organoid even in the presence of LPS. Similar effects of a monocyte-mediated immune tolerance have been reported in animal models of sepsis and clinical studies^62^. Thus, the liver organoid confirms the importance of these processes for hepatocellular functions. *In vivo* models represent the integrated systemic response to sepsis. Nevertheless, they also possess technical limitations. Sophisticated technology, i.e. intravital microscopy is needed for real-time analysis of immune cells interacting with the organ. In addition, the isolation of specific cell types in response to stimulation/intervention for subsequent analyses or even subculture is complex. The risk of a potential bias provoked by time-consuming tissue resection and digestion is increased. The modular structure of the liver organoid allows studying the role of individual cell types, stimuli and factors involved in the specific response. In this context and in contrast to the *in vivo* situation of animal models where e.g. depletion of HSCs is not possible, organoid models offer the advantage of studying the function of specific cell types by omitting or including them within a physiological tissue environment. In principle, also genetically modified cells/cell types can be embedded in a cellular “wild type” environment to study specific gene functions in the course of a simulated pathogenesis. From the practical point of view, the multi-layered assembly of the organoid model allows an easy disassembly of the different layers of the organoid and parallel isolation and analysis of cells from separated vascular and hepatic layers. Moreover, in contrast to the restrictions given in respect to the number of time points for sample collection from severely injured mice due to limitations of repeated blood drawings, the organoid paves the way for analysis of close-meshed trajectories in a more detailed manner. We are aware of the structural limitations of the liver model, i.e. the distance of 400 μm medium-filled space between the vascular and hepatic cell layer in contrast to the Disse space were cell layers are much closer to each other. However, our data demonstrate a selective barrier function of the vascular layer and that the hepatic layer responds to migrating monocytes. In the vascular layer macrophages are also interspersed between endothelial cells. In this setting small gaps at the contact zones between these two cell types are formed. Although this is not a substitution of endothelial fenestrae this cell layer allows passage of larger molecules from the vascular to the hepatic layer. This is demonstrated by action of cytokines that originate from secretion at the vascular compartment of the organoid and the observed active migration of monocytes to the hepatic cell layer. Thus, the liver organoid model presented herein demonstrates the impact and relevance of the cellular crosstalk between major cell types of the liver in the context of inflammation. In this first proof-of-concept study this model system presents itself highly suitable to investigate inflammation-associated liver dysfunction. More detailed follow-up studies using primary human cells are required to further confirm transferability to human sepsis conditions. Furthermore, it is of certain interest to investigate whether culture of primary hepatocytes within biochip organoids prevents loss of the polarized hepatocyte-phenotype and cell dedifferentiation observed in normal cell culture.

## Material and Methods

### Biochips

MOTiF biochips were made from polystyrol (PS) and obtained from microfluidic ChipShop GmbH (Jena, Germany). Biochips were manufactured as described previously^18^. Briefly, chips were made by injection molding. Within the Biochip an area of 1,1 cm^2^ is available for cell culture. The height of the vascular chamber is 700 μm, the hepatic chamber’s height is 400 μm. The width of the afferent and efferent channels is 0,8 mm and 2 mm, the height of these channels is 0,6 mm and 0,4 mm, respectively. The medium volume of the upper chamber including the channel system is 220 μl. The lower chamber volume including channel system housing the hepatic layer is 120 μl. A 12 μm thick PET membrane with a pore diameter of 8 μm and a pore density of 1 × 10^5^ pores/cm^2^ (TRAKETCH Sabeu, Radeberg, Germany) was integrated. Chips and channels structures were sealed on top and bottom side with an extruded 140 μm thick PS film using a low-temperature proprietary bonding method. Gas permeable silicon tubing was used for perfusion allowing oxygen equilibration during experimentation. In addition, the biochips owe a high re-diffusion of oxygen through the PS bulk material and 140 μm thin PS sealing films. No shortcoming of oxygen inside the chambers could be observed at the vascular or hepatic cell layer during culture (*SI Appendix*, Fig. S8A,B). Oxygen saturation was measured as previously described^17^. Ramping structures have been introduced into the chip bulk to avoid unfavorable flow conditions and trapping of stationary bubbles. Bubble formation was reduced by oxygen plasma treatment for hydrophilization of the whole chip surface and perfusion medium was stirred and equilibrated overnight under perfusion conditions before use.

### Oxygen measurement

Oxygen sensors were applied via spray coating at the inlet and outlet of each chamber, allowing online detection of oxygen consumption of cultivated cells. These sensors are based on dynamic quenching principle of luminescence by molecular oxygen and allow contactless measurements of oxygen via frame positioned polymer fibers. Read-out and data acquisition were accomplished by a commercially available oxygen meter (Firesting, Pyroscience, Aachen, Germany).

### Cell culture and TLR-Stimulation

*HepaRG hepatocytes:* HepaRG cells were obtained from Biopredic International (Rennes, France). They were seeded at a density of 2,7 × 10^4^ cells/cm^2^ and cultured in William’s Medium E (Biochrom, Berlin, Germany) containing 10% (v/v) FCS (GIBCO, Darmstadt, Germany), 5 μg/ml insulin (Sigma-Aldrich, Steinheim, Germany), 2 mM glutamine (GIBCO), 5 × 10^−5^ M hydrocortisone-hemisuccinate (Sigma-Aldrich) and 100 U/ml Penicillin/100 μg/ml Streptomycin mixture (Pen/Strep) (GIBCO). The cells were cultured in a humidified cell incubator at 5% CO_2_ and 37 °C for 14 days before differentiation. Medium was renewed every 3–4 days. Cell differentiation was induced as described^63^ and cells were used up to 4 weeks. Primary human cells: primary cell donors were informed about the aim of the study and gave written informed consent. The study and experimental protocols used therein were approved by the ethics committee of the Jena University Hospital (assigned study number 3939-12/13). *Endothelial cells:* HUVECs were isolated from human umbilical cord veins (HUVEC) as described^64^. Experiments were performed with HUVEC cells cultured in Endothelial Cell Medium (ECM) (Promocell, Heidelberg, Germany) up to passage 4, seeded with a density of 1.3 × 10^5^ cells/cm^2^ and cultured up to 95% confluence before sub-cultured. *Stellate cells:* LX-2 stellate cells were kindly provided by Scott L. Friedman (Division of Liver Diseases, Mount Sinai School of Medicine, New York City, NY, USA). LX-2 cells were cultured in Dulbecco’s Minimum Essential Medium (DMEM) (Biochrom, Berlin, Germany) supplemented with 10% (v/v) FCS, 1 mM sodium pyruvate (GIBCO) and Pen/Strep and seeded with a density of 2.0 × 10^5^ cells/cm^2^. Cells were grown up to 95% confluence before sub-cultured. *Primary macrophages:* PBMCs of three different healthy donors were isolated by Ficoll density gradient centrifugation and seeded in 6- well dishes with a density of 1.0 × 10^6^ cells/cm^2^ in X-VIVO 15 medium (Lonza, Cologne, Germany) supplemented with 10% (v/v) autologous human serum, 10 ng/ml human granulocyte macrophage colony-stimulating factor (GM-CSF) (PeproTech, Hamburg, Germany) and Pen/Strep. After 3 h incubation in a humidified cell incubator at 5% CO_2_ and 37 °C the cells were washed twice with *X-VIVO* 15 medium and subsequently used for liver organoid assembly. Typically, 5 × 10^5^ monocytes per ml whole blood were obtained. Liver organoids were assembled by staggered seeding of vascular and hepatic cell layers. In each sterilized biochip 3 × 10^5^ HUVEC’s and 1 × 10^5^ monocytes were mixed and seeded on top of the membrane in the upper chamber. HUVEC/monocytes were co-cultured for at least 5 days with a daily medium exchange in endothelial cell culture medium (ECM) supplemented 10 ng/ml epidermal growth factor, 90 μg/ml heparin, 2,8 μM hydrocortisone, endothelial cell growth supplement, 10 ng/ml GM-CSF to induce macrophage differentiation, 100 U/ml Penicillin/100 μg/ml Streptomycin and 10% (v/v) autologous human serum. Subsequently, 3 × 10^5^ differentiated HepaRG cells and 4 × 10^4^ LX-2 cells were seeded at the bottom sealing foil of the biochip in each bottom chamber and cultured for 24 hours in DMSO-free William’s Medium E (Biochrom, Berlin, Germany) hepatocyte growth medium containing 50 μM hydrocortisone, 10% (v/v) FBS containing, 5 μg/ml insulin, 2 mM glutamine and 100 U/ml Penicillin/100 μg/ml Streptomycin prior to experimental use. Endothelial growth medium was used in the upper biochip chamber and hepatocyte growth medium in the lower biochip chamber as specific cell culture media during native liver organoid culture and TLR stimulation experiments for the vascular and hepatic cell layers, respectively. TLR agonist stimulation was performed by medium exchange of the lower cavity chamber with hepatocyte growth medium supplemented with 100 ng/ml lipopolysaccharide (LPS) from *E. coli* 026:B6 (Sigma Aldrich), 1 μg/ml Pam3CSK4 (InvivoGen, Toulouse, France) or 5 μM ODN2006 (InvivoGen). Medium containing TLR agonists was renewed every 24 h. Organoid assembly and experimental setting is shown in Supplementary Fig. 1A.

### Monocyte migration assay

#### Primary monocytes

Monocytes were isolated from freshly isolated PBMCs of three different healthy donors using Dynabeads® CD14 Isolation Kit according to manufacturers protocol (Life Technologies, Darmstadt, Germany). Donors were informed about the aim of the study and gave written informed consent. The study was approved by the ethics committee of the Jena University Hospital. Typically, 5 × 10^5^ monocytes per ml whole blood were obtained. *THP-1 monocytes:* THP-1 cells were obtained from DSMZ (Braunschweig, Germany) and cultured in RPMI 1640 medium (GIBCO) supplemented with 10% (v/v) FCS, 1 mM sodium pyruvate and Pen/Strep. A total of 1 × 10^6^ THP-1 or primary monocytes were suspended in 16,2 ml ECM and perfused for 60 min with a flow rate of 270 μl/min over the the endothelial cell layer. This flow rate corresponds to a relative shear stress of 3 dyn/cm^2^ at the vascular cell layer.

After perfusion non-adherent cells were removed by perfusion of endothelial layer with cell free ECM medium for further 10 min with a flow rate of 270 μl/min. Subsequently, liver organoids were cultured for the indicated time periods under static conditions and observed effects compared to statically cultured controls without perfusion.

### Analysis of CYP3A4 metabolite formation

Liver organoids were cultured for 72h in absence or presence of LPS, respectively. Medium was exchanged every 24 hours. Subsequently, liver organoids were incubated for 6 h with serum-free hepatocyte culture medium containing Midazolam (Rotexmedica, Trittau, Germany), provided as an aqueous solution at 13.8 mM (5 mg/ml) and diluted to a final concentration of 3 μM. After protein precipitation and concentration, samples were analyzed using an LC-MS/MS system consisting of an ABSciex QTrap 4000 tandem mass spectrometer (Darmstadt, Germany) equipped with a Turbo V ion spray source and coupled to a LC-20 liquid chromatography system (Shimadzu, Jena, Germany). Separation was performed on a ZORBAX Eclipse XDBC18 column (4.6 × 150 mm, 5 μm) from Agilent (Böblingen, Germany) using a gradient program with 50 mM ammonium formate buffer plus 0.75% formic acid (eluent A) and acetonitrile (eluent B). The mass spectrometer was operated in scheduled multiple reaction monitoring (MRM) mode using the target transition *m/z* 342 to 324 for quantification of 1-OH-midazolam. Instrument control, data acquisition, and data evaluation were performed using Analyst software 1.6.2 (ABSciex, Darmstadt, Germany).

### Immunofluorescence staining and CDF-DA assay

For staining of cells of the hepatic cell layer the sealing foil of the biochip (serving as cell substrate) was carefully removed with a scalpel. For staining of cells from the vascular layer the suspended membrane was similarly removed. Cells were then fixed with 4% paraformaldehyde for 10 min at room temperature (RT). Staining was done with antibodies against: MRP-2, PECAM-1 (Cell Signaling, Leiden, The Netherlands), von Willebrand factor, VE-Cadherin (BD Biosciences), ApoB (Santa Cruz, Heidelberg, Germany), ZO-1 (Life Technologies, Karlsruhe, Germany), CYP3A4 (Merck-Millipore, Schwalbach, Germany) CD163 (Biolegend, United Kingdom), CD197 (BD BioScience), CD68 (Santa Cruz Biotechnology, Heidelberg, Germany) and secondary antibodies goat-anti-mouse-Cy3, goat-anti-rabbit-Cy5 (Dianova, Hamburg, Germany), goat-anti-rabbit-AlexaFluor488 and DAPI (Life Technologies). Samples were embedded into fluorescent mounting medium (Dako, Hamburg, Germany). MRP-2 activity analysis was performed by incubation of HepaRG cell layers in serum free Williams E medium (GIBCO) containing 5 μM 5(6)-Carboxy-2**′**,7′-dichlorofluorescein diacetate (CD-FDA) (Sigma-Aldrich) at 37 °C for 15 min. Subsequently, imaging was performed on an AXIO Observer Z1 fluorescence microscope with Apotome 2 extension (Carl Zeiss AG, Jena, Germany). Image analysis was done with ImageJ2 software.

### FACS analysis

Cells of the vascular or hepatic layer were separately detached and collected from the biochip using 4 mg/ml Lidocaine and 5 mM EDTA (Sigma Aldrich) in PBS (Lonza). Flow cytometric analysis of ECs was performed using antibodies CD54-AF647 and CD106-APC (Biolegend, Fell, Germany). Transmigrated primary monocytes were collected in the lower chamber of the biochip. Monocytes were stained with CD45-FITC antibody (Immunotools, Friesoythe, Germany). Viability of cells from the hepatic layer was assessed by staining with Annexin V (conjugated to APC, BD Bioscience) for apoptotic cells and 7AAD (BD Bioscience) for dead cells. Flow cytometry was performed on a BD FACS-Canto II (BD Biosciences, Germany) with FACSDiva software and analyzed using FlowJo X software (FlowJo LLC, Ashland, OR, USA).

### Cytometric bead array (CBA)

Supernatants were collected after indicated time periods and immediately frozen at −80 °C. Cytokines were detected using CBA assay (BD Biosciences) according to the manufacturer’s protocol. Enhanced sensitivity flex set was used for measurement of TNFα and IL-1β release. Secretion of IL-6, IL-10, sICAM-1 and sVCAM-1 was analyzed using standard CBA flex sets. Analysis was performed on a BD FACS-Canto II cytometer with FACSDiva software. Data analysis was performed using FCAP Array V3 software (Softflow, Pecs, Hungary).

### Lactate, Glucose, Albumin, Urea, ASAT, ALAT, GLDH and LDH measurements

The respective parameters were measured in cell culture supernatants using the Abbott Architect ci8200 Integrated System (Abbott Laboratories, Abbott Park, IL, USA) according to the manufacturer’s protocol.

### Peritoneal contamination and infection in mice

Severe sepsis was induced using a peritoneal contamination and infection model by administration of standardized fecal slurry as described previously^44^ and approved by the local animal welfare committee (TLLV, 02-037/12). Female C57Bl/6 mice (aged 12–14 weeks, n = 55) undergoing sepsis were monitored and resuscitated for four days (25 μL/g B.W. balanced saline solution, twice a day, s.c.). Starting six hours following insult, mice underwent antibiotic rescue (meropenem, 25 μg/g B.W., twice a day for four days, s.c.). Overall survival rate (21 days, unfavorable outcome was only observed in between first 72 hours) accounted to 74%. At time points indicated randomly selected mice were removed (at least five animals/time points), deeply anaesthetized for subsequent preparation of liver tissue and plasma samples. Parameters of organ (dys)function were determined using a Fuji Dri Chem 3000i analyzer according manufacturer’s instructions.

RNA was isolated from freshly frozen tissue (25–30mg) applying RNeasy Mini Kit (QIAGEN, Hilden Germany). Via reverse transcription 1μg RNA was used to convert the mRNA fraction into single strand cDNA (Thermo Scientific, Germany). Using Rotorgene Q System (QIAGEN, Hilden Germany) expression rate of CYP3A11(fw: 5′-agc agg gat gga cct gg-3′; rv: 5′-cgg tag agg agc acc aa-3′) and Mrp2 (fw: 5′-aac ttg ggt tgc tcc atg a-3′; rv: 5′-cag gac cag gat ttt gga ttt-3′) were tested and normalized to GusB (fw: 5′-gaa acc cgc cgc ata tta c-3′; rv: 5′-ccc cag gtc tgc atc ata tt-3′) according to the method of Pfaffl^65^. All tests were performed according to manufacturer’s instructions. In parallel, freshly frozen tissue was equally used to determine enzyme activity of CYP3A family using the ethylmorphine N-demethylation model reaction as previously described^66^.

### Statistics

For each experiment shown at least three independent experiments have been performed. Statistical analysis has been performed with GraphPad Prism 6.05 (GraphPad Software, La Jolla, CA, USA). For analysis of statistical significance student’s t-test, one-way ANOVA with Dunnett’s multiple comparisons or two-way ANOVA with Dunnett’s multiple comparisons test have been used as indicated.

## Additional Information

**How to cite this article**: Gröger, M. *et al.* Monocyte-induced recovery of inflammation-associated hepatocellular dysfunction in a biochip-based human liver model. *Sci. Rep.* **6**, 21868; doi: 10.1038/srep21868 (2016).

## Supplementary Material

Supplementary Information
