# Corrigendum: Monocyte-induced recovery of inflammation-associated hepatocellular dysfunction in a biochip-based human liver model

**DOI:** 10.1038/srep46988

**Published:** 2018-05-14

**Authors:** Marko Gröger, Knut Rennert, Benjamin Giszas, Elisabeth Weiß, Julia Dinger, Harald Funke, Michael Kiehntopf, Frank T. Peters, Amelie Lupp, Michael Bauer, Ralf A. Claus, Otmar Huber, Alexander S. Mosig

Scientific Reports 6: Article number: 2186810.1038/srep21868; published online: 02 23 2016; updated: 05 14 2018

The Acknowledgements section in this Article is incomplete.

“We thank Torsten Mayr and Josef Ehgartner (Institute of Analytical Chemistry and Food Chemistry, Graz University of Technology, Graz, Austria) for providing oxygen sensors. We are grateful to the excellent technical work of Maria Franke, Margot Voigt and Daniela Remane. We thank the team of the Placenta Laboratory of the Jena University Hospital for supplying umbilical cords for HUVEC isolation. The authors would further like to acknowledge support of this work by 2011 VF 0005 grant of the Thüringer Aufbaubank. This work was further supported by the Federal Ministry of Education and Research (BMBF), Germany, FKZ: 01EO1002.”

should read:

“We thank Torsten Mayr and Josef Ehgartner (Institute of Analytical Chemistry and Food Chemistry, Graz University of Technology, Graz, Austria) for providing oxygen sensors. We are grateful to the excellent technical work of Maria Franke, Margot Voigt and Daniela Remane. We thank the team of the Placenta Laboratory of the Jena University Hospital for supplying umbilical cords for HUVEC isolation. The authors would further like to acknowledge support of this work by 2011 VF 0005 grant of the Thüringer Aufbaubank. This work was further supported by the Federal Ministry of Education and Research (BMBF), Germany, FKZ: 01EO1002. The authors further acknowledge support of this work by a grant from the German Federal Institute for Risk Assessment (grant no. 1328-511).”

