# Supplementary material for: Monocyte-induced recovery of inflammation-associated hepatocellular dysfunction in a biochip-based human liver model
Source: Sci Rep. 2016 Feb 23;6:21868. doi: 10.1038/srep21868 (PMC4763209; doi:10.1038/srep21868)
Supplement: Supplementary Information [file srep21868-s1.pdf]

Supplementary Material to Manuscript SREP-15-29162A

Monocyte-induced recovery of inflammation-associated hepatocellular dysfunction in a biochip-based human liver model

Authors:

Marko Gröger[a,f,1], Knut Rennert[a,1], Benjamin Giszas[b,g], Elisabeth Weiß[a], Julia Dinger[c], Harald Funke[d], Michael Kiehntopf[e,g], Frank T. Peters[c], Amelie Lupp[f], Michael Bauer[b,g], Ralf A. Claus [b,g], Otmar Huber[a,g], Alexander S. Mosig[a,g,2]

a) Institute of Biochemistry II, Jena University Hospital, 07743 Jena, Germany; b) Clinic of Anesthesiology and Intensive Care, Jena University Hospital, Jena 07747 Jena, Germany; c) Institute of Forensic Medicine, University Hospital Jena, 07743 Jena, Germany; d) Molecular Hemostaseology, Jena University Hospital, Jena, 07747 Jena, Germany; e) Department of Clinical Chemistry and Laboratory Medicine, Jena University Hospital, 07747 Jena, Germany; f) Institute of Pharmacology and Toxicology, Jena University Hospital, Jena; g) Center for Sepsis Control and Care, Jena University Hospital, Jena, 07747 Jena, Germany

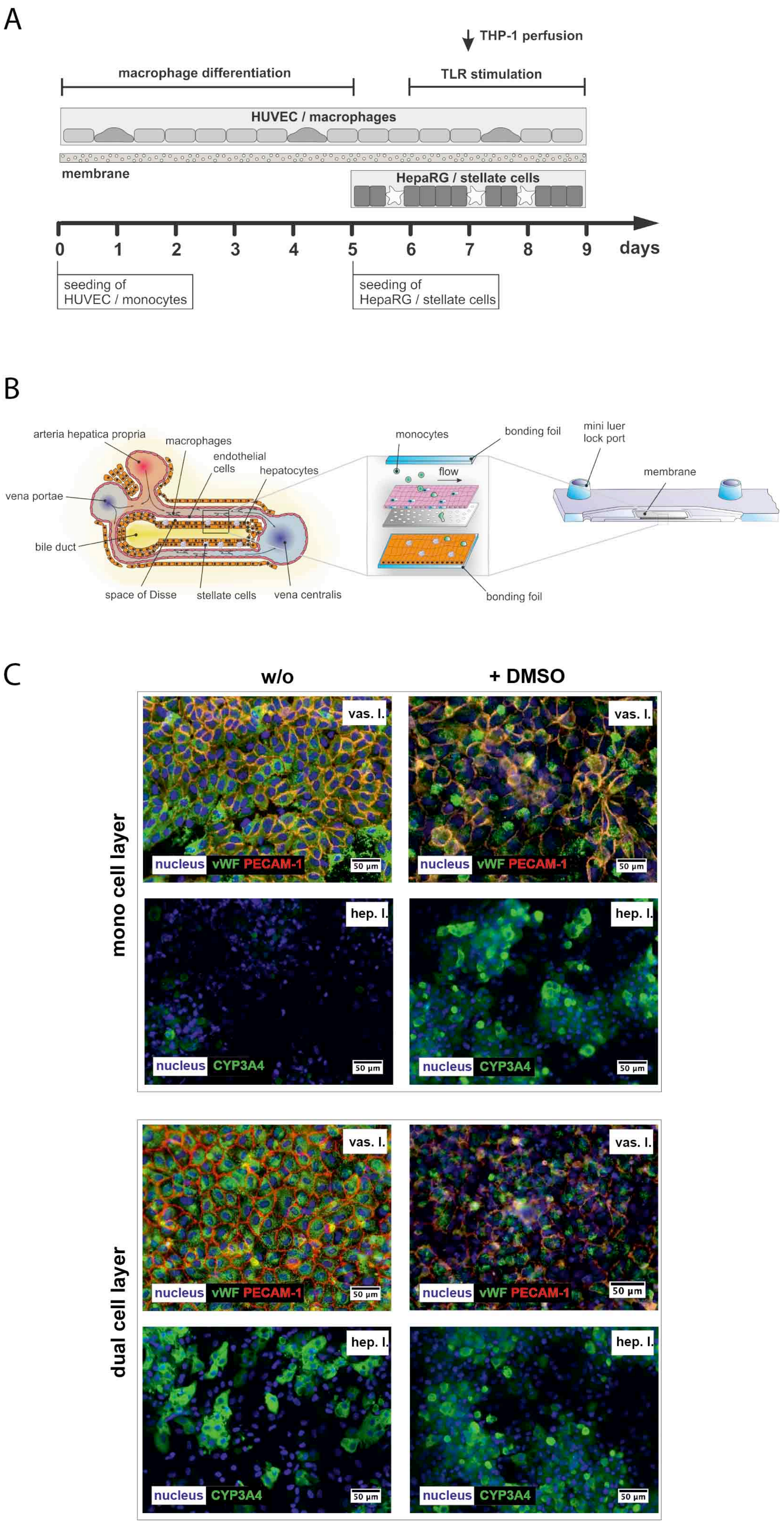
A
THP-1 perfusion
macrophage differentiation
TLR stimulation
HUVEC / macrophages
membrane
HepaRG / stellate cells
0 1 2 3 4 5 6 7 8 9 days
seeding of HUVEC / monocytes
seeding of HepaRG / stellate cells
B
arteria hepatica propria
macrophages
endothelial cells
hepatocytes
vena portae
bile duct
space of Disse
stellate cells
vena centralis
monocytes
bonding foil
flow
mini luer lock port
membrane
bonding foil
C
w/o
+ DMSO
mono cell layer
dual cell layer
vas. l.
hep. l.
nucleus vWF PECAM-1
nucleus CYP3A4
50 µm

Supplementary Figure 1. A) Timeline of organoid assembly and experimental setting. B) Principle structure of the human sinusoid and abstracted implementation as tissue layers in the MOTiF biochip. C) Effects of DMSO addition on expression of von Willebrand Factor (vWF, green) and PECAM-1 (red) on vascular layers (vas. l.), and CYP3A4 on hepatic layers (hep. l.) in mono cell layer and dual cell layer culture. Representative data out of five independent experiments are shown. Nuclei are stained with DAPI (blue).

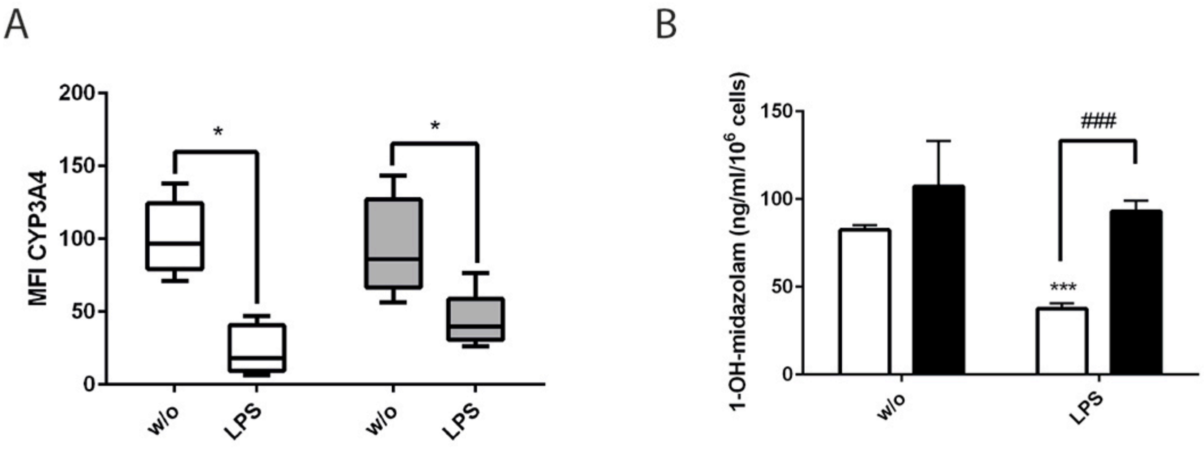

Supplementary Figure 2. A) Expression of CYP3A4 after 72 h of LPS treatment in mono-cell cultures of HepaRG cells (open bars) and HepaRG cells co-cultured with stellate cells (grey bars). Computational analyses of fluorescence intensities of at least 20 ROIs per condition and experiment (labeled as mean immunofluorescence intensity (MFI) of specific stainings of CYP3A4) of three independent experiments. B) Formation rate of 1-OH-midazolam in HepaRG mono-cell cultures (open bars) and in liver organoids (black bars) of three independent experiments. Statistical significance was calculated compared to untreated control (* $p < 0.05$, *** $p < 0.01$) or between indicated conditions (## $p < 0.01$, ## $p < 0.001$) using student's t-test.

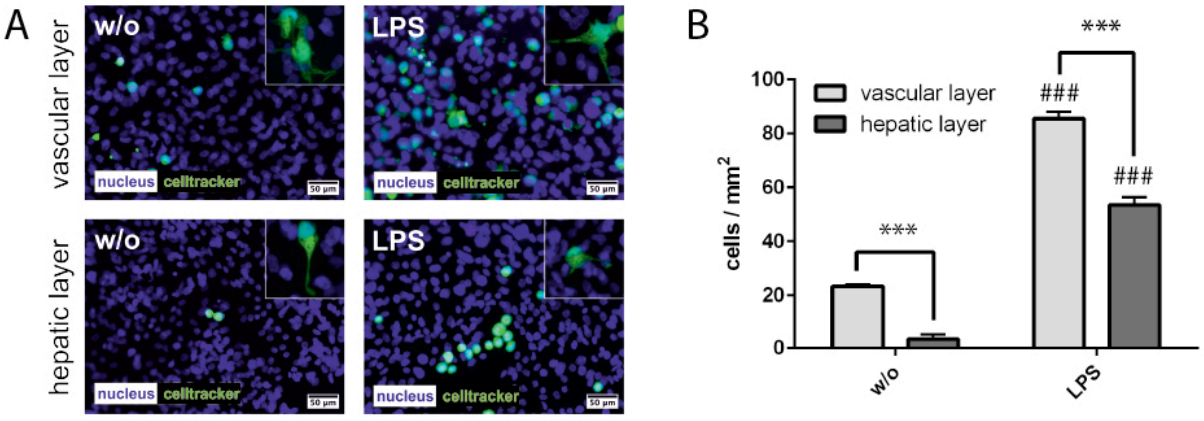

Supplementary Figure 3. A) Flow-based adhesion and migration assay of THP-1 monocytes stained with Celltracker® Green at the vascular and hepatic layer of liver organoids in absence (w/o) of LPS or liver organoids 24 h pre-stimulated with LPS. Nuclei are stained with DAPI (blue). Representative data out of three independent experiments are shown. B) Number of adhesive and transmigrated THP-1 monocytes at the vascular or hepatic layer 48 h post monocyte perfusion. Statistical significance was calculated between indicated conditions (*** $p < 0.001$) or compared to the corresponding condition without LPS treatment (### $p < 0.001$) using student's t-test. Results of three independent experiments are shown.

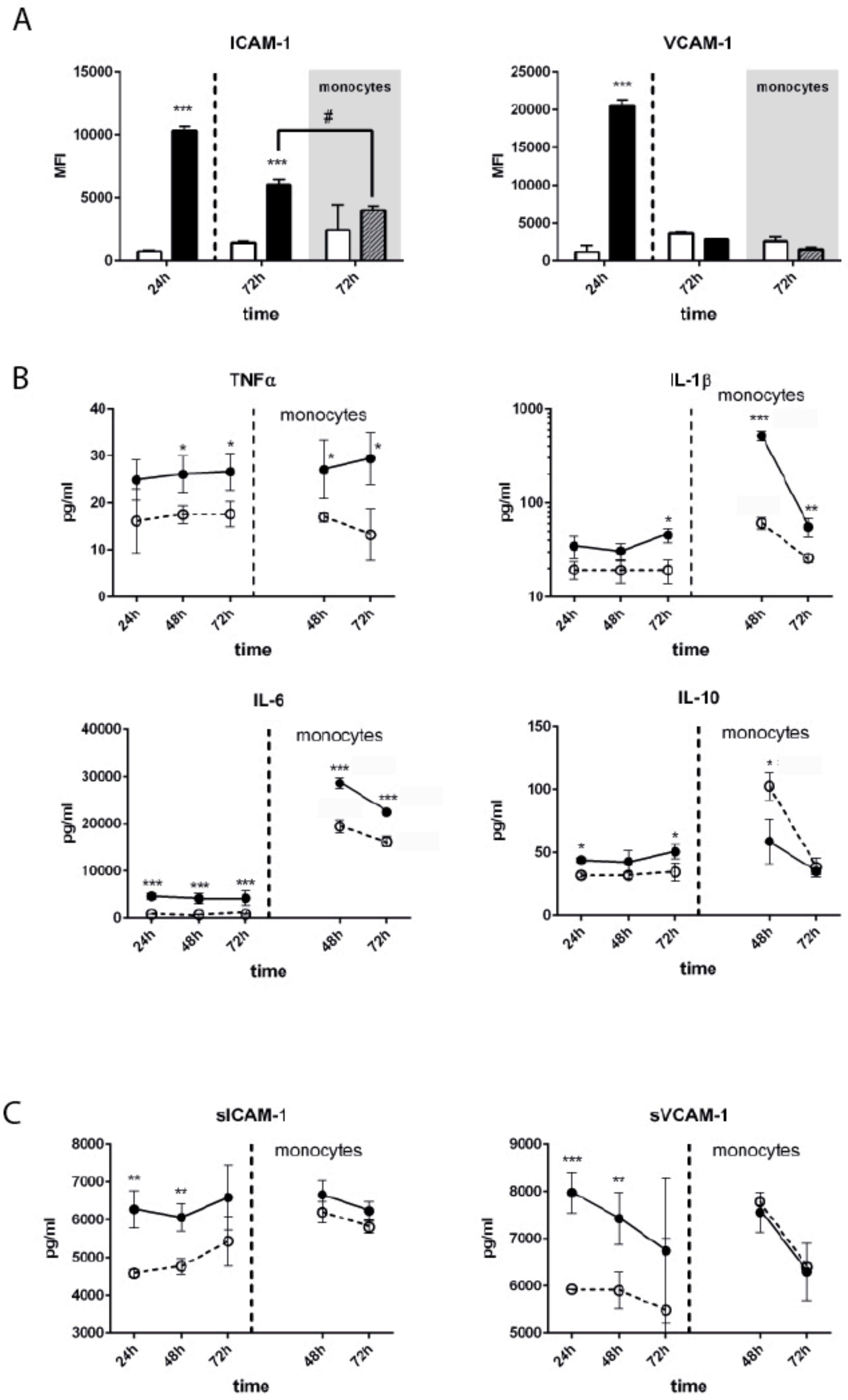

A
ICAM-1
15000
10000
5000
0
MFI
monocytes
#
***
***
24h
72h
72h
time
VCAM-1
25000
20000
15000
10000
5000
0
MFI
monocytes
***
24h
72h
72h
time
B
TNFα
40
30
20
10
0
pg/ml
monocytes
*
*
*
*
24h
48h
72h
48h
72h
time
IL-1β
1000
100
10
pg/ml
monocytes
***
**
*
24h
48h
72h
48h
72h
time
IL-6
40000
30000
20000
10000
0
pg/ml
monocytes
***
***
***
***
***
24h
48h
72h
48h
72h
time
IL-10
150
100
50
0
pg/ml
monocytes
*
*
*
24h
48h
72h
48h
72h
time
C
sICAM-1
8000
7000
6000
5000
4000
3000
pg/ml
monocytes
**
**
24h
48h
72h
48h
72h
time
sVCAM-1
9000
8000
7000
6000
5000
pg/ml
monocytes
***
**
24h
48h
72h
48h
72h
time

Supplementary Figure 4. A) Expression of endothelial cell adhesion molecules (CAMs) and release of soluble CAMs and cytokines by THP-1 monocytes. A) Surface expression of CAMs of untreated (open bars) and LPS-treated liver organoids (black bars) after 24 h and 72 h without THP-1 monocyte perfusion (white background) and with THP-1 monocyte perfusion (grey background). Flow cytometric analyses of mean fluorescence intensities (MFI) of fluorescence-labeled antibodies directed against endothelial ICAM and VCAM, respectively. Statistical significance was calculated between untreated and LPS treated liver organoids of identical time points (*** $p < 0.001$) or between indicated conditions (# $p < 0.05$) using student's t-test. B) CBA assay of released sICAM and sVCAM in response to THP-1 perfusion and adhesion. C) Secretion of IL-1β, IL-6, TNFα and IL-10 in response to THP-1 perfusion and adhesion. Liver organoids were untreated (dashed line) or stimulated with LPS (solid line), without THP-1 monocyte perfusion (left from vertical dashed line) or with THP-1 monocyte perfusion (right from vertical dashed line, monocytes). A-C) Statistical significance was calculated between untreated and LPS-treated liver organoids of identical time points and perfusion conditions (* $p < 0.05$, ** $p < 0.01$, *** $p < 0.001$) using student's t-test. Results of three independent experiments are shown. B-C) Data of liver organoids without monocyte perfusion are identical to data used in Fig. 4.

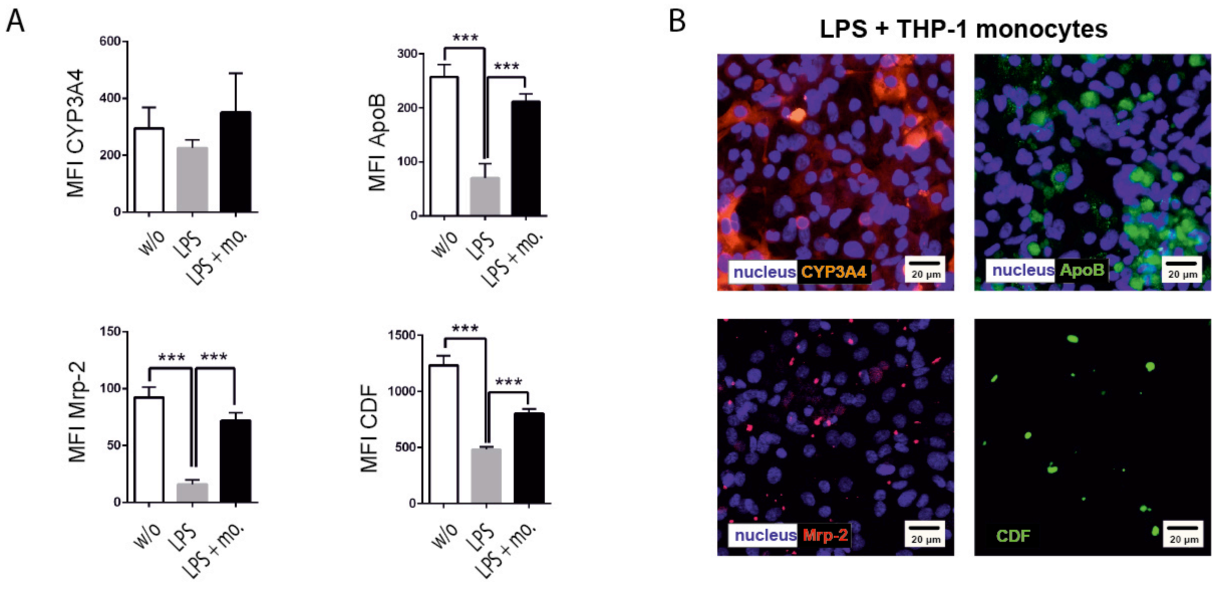

Supplementary Figure 5 A) Computational analyses of fluorescence intensities of hepatocyte proteins without stimulation (w/o) and in presence of 100 ng/ml LPS (LPS) or 100 ng LPS and THP-1 monocytes (LPS + mo.) of at least 20 ROI per tested condition (labeled as mean immunofluorescence intensity (MFI) of specific staining of the respective protein) of three independent experiments using random field analysis of the hepatic layer. Statistical significance was calculated between indicated conditions using student's t-test (* $p < 0.05$, ** $p < 0.01$, $p < 0.001$). B) Liver organoids treated with LPS in presence of invading THP-1 monocytes. Immunostainings of the hepatic layer for CYP3A4 (red), ApoB (green), MRP-2 (red) and detection of CDF secretion (green). Nuclei are stained with DAPI (blue). Representative results of three independent experiments are shown.

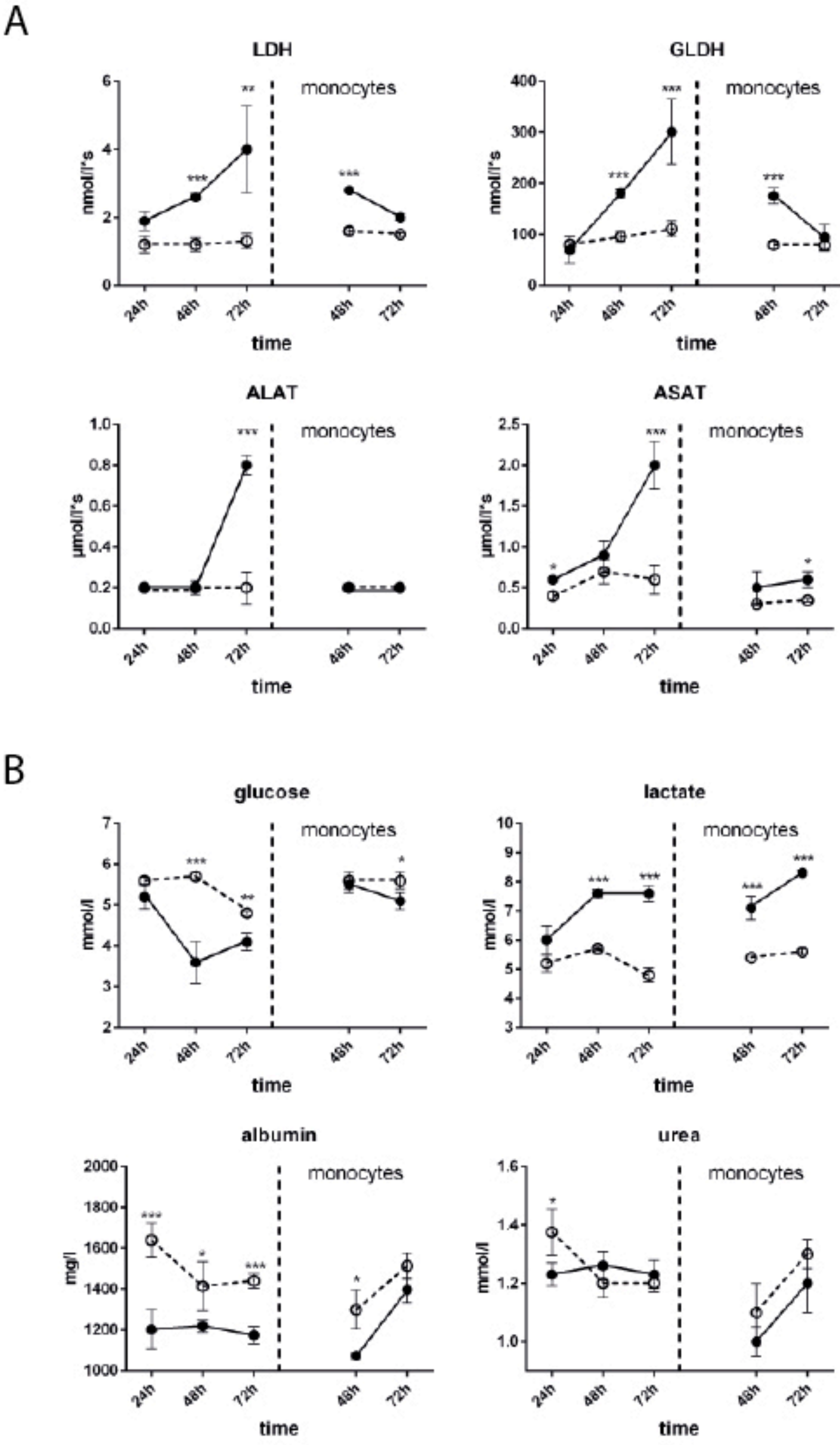

A
LDH
nmol/l*s
monocytes
GLDH
ALAT
µmol/l*s
ASAT
24h
48h
72h
time
B
glucose
mmol/l
lactate
albumin
mg/l
urea

Supplementary Figure 6 A-B) Release of intracellular enzymes and metabolic activity of the liver organoid. Liver organoids were untreated (dashed line) or stimulated with LPS (solid line), without monocyte perfusion (left from vertical dashed line) or with THP-1 monocyte perfusion (monocytes, right from vertical dashed line). A) Release of lactate-dehydrogenase (LDH), glutamate-dehydrogenase (GLDH), aspartate-transaminase (ASAT) and alanine-transaminase (ALAT). B) Changes in glucose, lactate, albumin and urea levels. Statistical significance was calculated compared to untreated control (* $p < 0.05$, ** $p < 0.01$, *** $p < 0.001$) at the corresponding time points with similar perfusion conditions using student's t-test. A-B) Data of liver organoids without monocyte perfusion are identical to data used in Fig. 6.

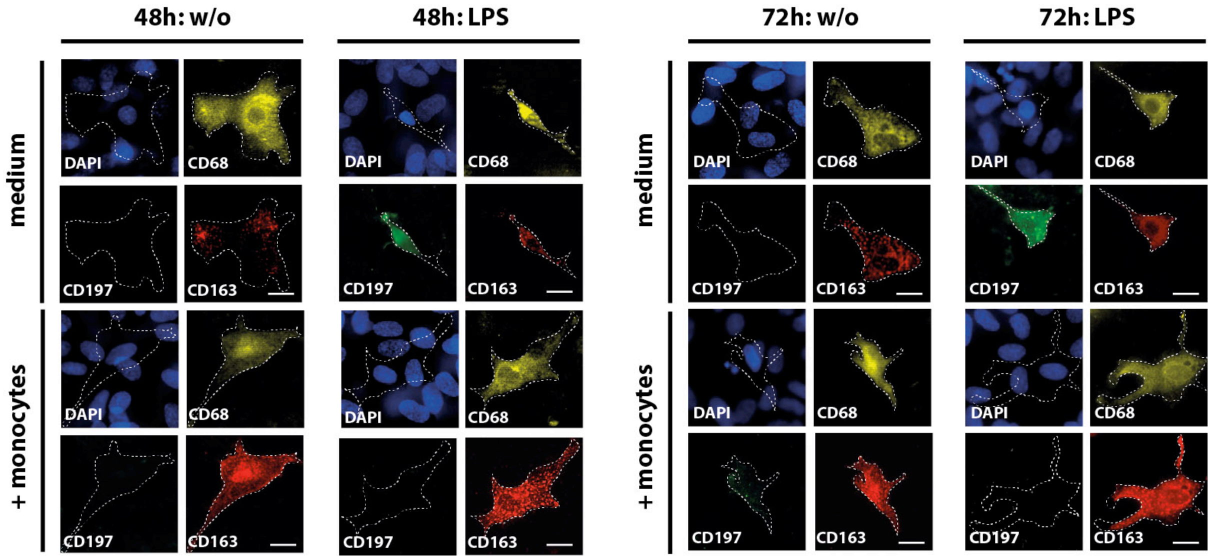

Supplementary Figure 7. Liver organoids were cultured for 48 h or 72 h. Untreated (w/o) or LPS-stimulated (LPS) organoids were perfused with medium (medium) or medium containing primary monocytes (+ monocytes). Macrophages residing in the vascular layer of the liver organoid were immunostained for macrophage marker protein CD68 (yellow), M1-polarization marker protein CD197 (green) and M2-polarization marker protein CD163 (red). Nuclei were stained with DAPI (blue). Cell borders are indicated by dashed lines. Scale bar 10µm.

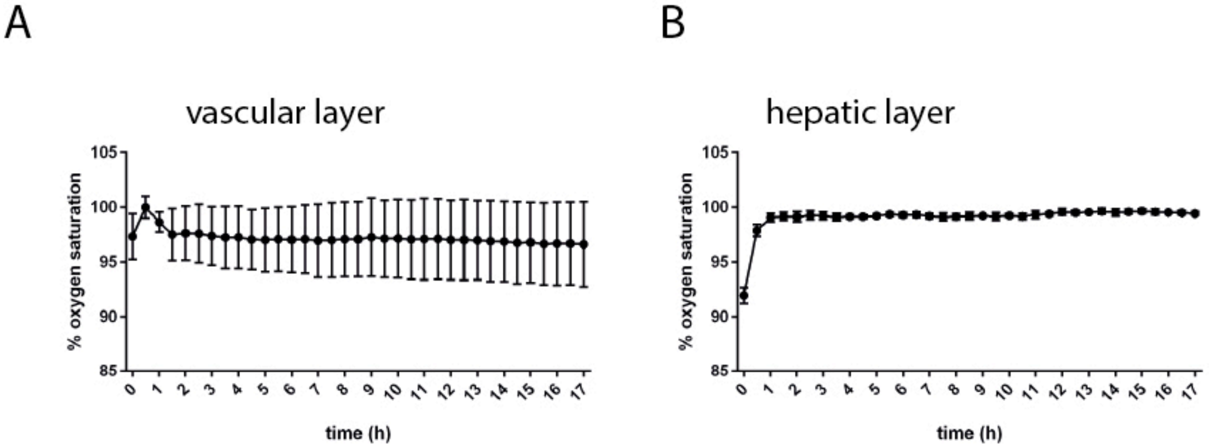

Supplementary Figure 8. A-B) Oxygen level in the A) vascular and B) hepatic chamber within MOTiF biochips during liver organoid culture. A 100% oxygen saturation corresponds to 20,942 % atmospheric O_2. Shown is the mean of percent oxygen saturation of three independent measurements with respective standard deviation.
